# Cerebrovascular Disease Mortality Trends in Brazil: An In-Depth Joinpoint Analysis

**DOI:** 10.7759/cureus.45845

**Published:** 2023-09-24

**Authors:** Billy McBenedict, Wilhelmina N Hauwanga, Aisha Elamin, Filagot D Eshete, Noama El Husseini, Abdullah A El Ghazzawi, Vaishvik K Patel, Bruno L Pessôa, Julio Tolentino, Evandro T Mesquita

**Affiliations:** 1 General and Specialized Surgery, Universidade Federal Fluminense, Niteroi, BRA; 2 General and Specialized Surgery, Universidade Federal do Estado do Rio de Janeiro, Rio de Janeiro, BRA; 3 Medicine, National University, Khartoum, SDN; 4 General Surgery, Jimma University, Jimma, ETH; 5 Medicine, Beirut Arab University, Beirut, LBN; 6 Medicine, St. George’s University, West Indies, GRD; 7 Clinical Medicine, Universidade Federal Fluminense, Niteroi, BRA

**Keywords:** brazil, joinpoint analysis, age-adjusted mortality rate, stroke, cerebrovascular disease

## Abstract

Background

Cerebrovascular disease is the second leading cause of death and the third leading cause of disability following heart disease. In 2019, there were over 101 million people living with a stroke and 12.2 million incidents of stroke globally. For the past three decades, stroke has remained the leading cause of death in Brazil, causing over 100,000 fatalities annually, along with numerous functional impairments among those who survive. The Brazilian healthcare system has witnessed notable advancements in the last decade, including the establishment of additional hospitals and a rise in the count of healthcare professionals specializing in cardiovascular and neurological surgery. However, there exists a gap in the research landscape for continuous comprehensive studies aimed at exploring the evolving mortality rates related to cerebrovascular diseases, of which the last one included data up to 2019. This study aimed to address this gap by meticulously analyzing the trends in cerebrovascular disease mortality in Brazil from 2000 to 2021, for the variables age, sex, state of residence, and geographic region.

Methods

This is a descriptive, ecological, and time series study. Nationwide data for annual cerebrovascular mortality from Brazil were used for the period 2000-2021. Age-adjusted mortality rates (AAMRs) by direct standardization, encompassing people above 20 years of age, were calculated and expressed per 100,000 persons. Mortality trends were assessed using joinpoint regression analysis by calculating the annual percentage change (APC) and its corresponding 95% confidence interval (CI) across categories of age, sex, and state and region of residence.

Results

The mortality rates decreased for the sex categories over the analyzed years. The AAMR for the categories decreased as follows: males and females (95 deaths/100,000 to 52 deaths/100,000 inhabitants), males (108 deaths/100,000 to 63 deaths/100,000 inhabitants), and females (83 deaths/100,000 to 44 deaths/100,000 inhabitants). The most substantial reduction in AAMR for males occurred in the 30-39-year age group (APC: -4.10), while the smallest decline was observed in the 20-29-year age group (APC: -1.44). All five macro-regions demonstrated statistically significant and downward AAPC values in mortality rates. The south and midwest regions decreased at a stable rate, as denoted by the same APC and AAPC values (-4.05 and -3.11, respectively). The north and northeast regions exhibited an increase in AAMR, followed by a decrease (APC: 0.68 to -1.42 and 2.63 to -2.35, respectively).

Conclusions

Our comprehensive analysis revealed a downward trend in cerebrovascular disease mortality rates across diverse demographic groups and macro-regions. Females experienced a more substantial reduction compared to males. Despite higher mortality rates among individuals aged 50 and above, all age groups displayed a marked decrease. The continuous decline can be attributed to policy interventions aimed at enhancing healthcare delivery, increased awareness, and healthier diets and lifestyles. With regard to the macro-regions, the regions in the southern zone demonstrated a more significant decrease as compared to the northern part. In Brazil, a more significant decline in cerebrovascular disease mortality rates could be achieved through increased focus on prevention measures and efforts toward mitigating disparities and inequalities between macro-regions.

## Introduction

Cerebrovascular disease encompasses all disorders in which an area of the brain is temporarily or permanently affected by hemorrhage or ischemia due to cerebral blood vessels undergoing a pathological process. The lack of a constant supply of oxygen and nutrients leads to brain cell damage and death within a few minutes. Since brain cells cannot regenerate, cell death results in physical, cognitive, and mental disabilities. Common cerebrovascular diseases include ischemic stroke, hemorrhagic stroke, and transient ischemic attacks. Ischemic strokes occur when an artery is obstructed by plaque or an embolus in either the carotid or vertebral arteries, whereas hemorrhagic strokes are often associated with blood vessel ruptures, commonly triggered by hypertension, which results in tissue damage [1]. Transient ischemic attacks are brief blockages of brain arteries, resolving in less than 24 hours and before any permanent damage occurs [1].

The various conditions that fall under the category of cerebrovascular disease are delineated within the WHO classification using ICD 10 codes I60-I69, and these include nontraumatic subarachnoid, intracerebral, and intracranial hemorrhage, cerebral infarction, and occlusion and stenosis of the cerebral and precerebral arteries. The most important modifiable risk factors for cerebrovascular disease are hypertension, hyperlipidemia, hormone replacement therapy, diabetes mellitus, tobacco use, and atrial fibrillation. The non-modifiable risk factors include advanced age, low birth weight, genetic factors, and having an African-American or Hispanic background [2].

Cerebrovascular disease is more common in men than women and is the second leading cause of death and the third leading cause of disability following heart disease [3]. Stroke is the most common type of cerebrovascular disease. In 2019, there were over 101 million people living with a stroke and 12.2 million incidents of stroke globally [4]. It also accounted for 6.55 million deaths worldwide and 143.23 million disability-adjusted life years (DALYs) [4]. The incidence of stroke is declining in many developed countries due to better control of modifiable risk factors, increased use of preventive treatment, and access to quality stroke care. On the other hand, the absolute number of strokes continues to increase because of the aging population [5]. Despite its increased prevalence among older adults, cerebrovascular disease can manifest at any stage of life.

Brazil is the largest country in South America and the seventh most populous nation in the world. For the past three decades, stroke has remained the leading cause of death in Brazil, causing over 100,000 fatalities annually, along with numerous functional impairments among those who survive [6]. The nation is geographically divided into five regions: north encompassing states such as Acre, Rondônia, Amazonas, Pará, Tocantins, Roraima, and Amapá; northeast including states such as Maranhão, Piauí, Ceará, Rio Grande do Norte, Paraíba, Alagoas, Sergipe, Bahia, and Pernambuco; southeast consisting of São Paulo, Espirito Santo, Minas Gerais, and Rio de Janeiro; south comprising states such as Paraná, Santa Catarina, and Rio Grande do Sul; and midwest consisting of Goias, Mato Grosso, and Mato Grosso do Sul [7]. The Brazilian healthcare system has witnessed notable advancements in the last decade, including the establishment of additional hospitals and a rise in the count of healthcare professionals specializing in cardiovascular and neurological surgery [8]. Despite these improvements, there exists a gap in the research landscape as there is a need for continuous comprehensive studies aimed at exploring the evolving mortality rates related to cerebrovascular diseases. This study aims to address this gap by meticulously analyzing the trends in cerebrovascular disease mortality rates in Brazil from 2000 to 2021, taking into account factors such as age, sex, state of residence, and geographic region.

## Materials and methods

Study design and data collection

This is an observational, descriptive, and ecological time-series study, employing a quantitative and descriptive analysis using secondary data. For this nationwide study, we used data for annual cerebrovascular mortality from Brazil to describe cerebrovascular disease mortality trends for the period 2000-2021. Data on individuals who died of cerebrovascular disease, according to the International Classification of Diseases (ICD) Tenth Revision codes I60-I69, were obtained from the Brazilian Hospital Information System (DATASUS) under the Sistema Único de Saúde (SUS), a Brazilian Unified Health System. Information was collected under the section "Sistema de Informações sobre Mortalidade (SIM)," which is a mortality information system used to regularly obtain data on mortality in Brazil based on death certificates collected by the State Health Departments. DATASUS compiles data from all SUS-reimbursed hospitalizations, covering about 80% of the population, and it stands as a testament to Brazil's successful experience in the domain of health information management, a project spearheaded by the Federal Government of Brazil [9]. In the case of deaths, it is mandatory to report and record all deaths, especially since is is used as an important index of health in Brazil.

Data analysis

Population Estimates and Mortality Rates

Information regarding the population/demographics of Brazil (counts based on sex, age, state of residence, and region of residence) were obtained from the population estimates provided by the Brazilian Institute of Geography and Statistics (IBGE), under the Demographic and Socioeconomic Information section. During the period under observation, the population of Brazil exhibited consistent growth, surging from 173,765,726 residents in the year 2000 to 213,317,639 inhabitants by the year 2021. The population of interest experienced growth from 103,167,559 inhabitants in the year 2000 to 153,748,438 inhabitants in the year 2021. We calculated cerebrovascular disease age-adjusted mortality rates (AAMRs) encompassing people above 20 years of age for the following categories: age-specific, sex-specific, and state and region of residence. Mortality rates were expressed per 100,000 persons. AAMRs were calculated by direct standardization, employing the Segi world standard population.

Time Series Analysis

Cerebrovascular disease mortality trends were estimated using joinpoint regression analysis. Joinpoint Regression Program (Version 5.0.2., May 2023) is a trend analysis software developed by the Statistical Research and Applications Branch of the National Cancer Institute (United States), for the analysis of data from the Surveillance Epidemiology and End Results Program (SEER). Joinpoint analysis was used to identify the best-fitting point, where a statistically significant change (called the “joinpoint”) had occurred and to determine the trends between joinpoints. The “grid search” method was selected. The test of significance used was the weighted Bayesian information criterion to determine the best-fitting combination of line segments and joinpoints [10]. The number of joinpoints ranged from “0” to “4.” Cerebrovascular disease mortality trends were assessed by calculating the annual percentage change (APC) and its corresponding 95% confidence interval (CI) between consecutive change points. Additionally, an average annual percentage change (AAPC) was computed as a summary of the trend analysis. This examination aimed to investigate variations in cerebrovascular disease mortality trends across categories, such as age, sex, and both the state and region of residence.

## Results

**Figure 1:**
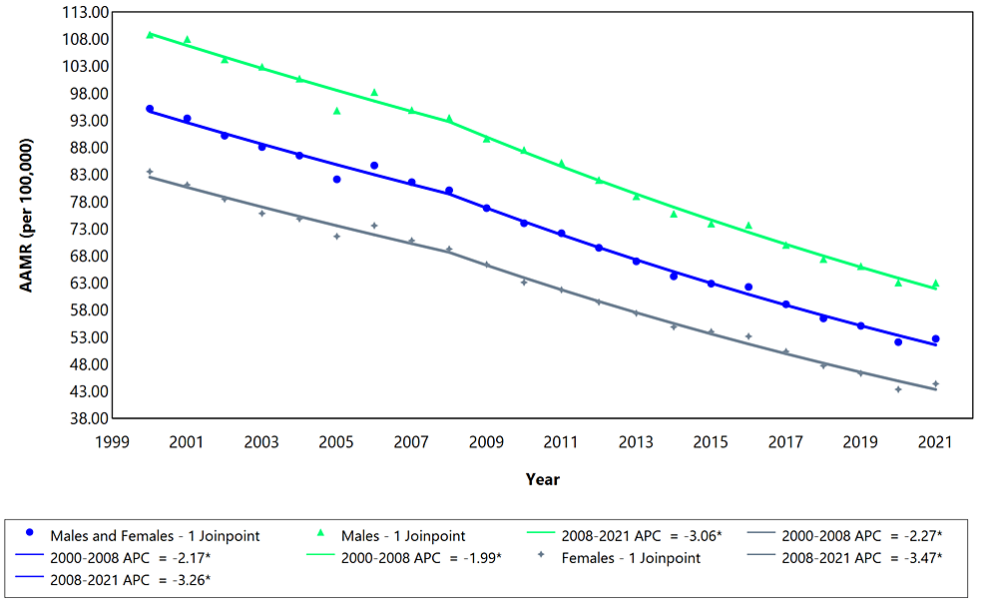
Cerebrovascular disease AAMR trend Joinpoint analysis for the variable sex during the period 2000-2021. *Significant at P < 0.05 level. AAMR = Age-adjusted mortality rate; APC = Annual percent change.

The cerebrovascular disease mortality rate trend was downward for the categories of males, females, and males and females combined, over the analyzed years, as demonstrated by the average annual percent change (AAPC, Table 1). All three categories had two segments each (2000-2008 and 2008-2021), with significant annual percent changes (APC) per segment, as shown in Table 1, and visually represented in Figure 1 below. In addition, the reduction in mortality was more pronounced among females than males (see AAPC values).

**Table 1:** Cerebrovascular disease AAMR trend joinpoint analysis for the variable sex during the period 2000-2021. *Significant at P < 0.05 level. AAMR = Age-adjusted mortality rate; AAPC = Average annual percent change; APC = Annual percent change.

Sex	Period (year)	APC (95% CI)	AAPC (95% CI)	Interpretation
Males and Females	2000-2008	-2.17* (-2.50 to -1.56)	-2.85* (-2.97 to -2.72)	Decrease
Males and Females	2008-2021	-3.26* (-3.55 to -3.07)		Decrease
Males	2000-2008	-1.99* (-2.35 to -1.27)	-2.65* (-2.80 to -2.51)	Decrease
Males	2008-2021	-3.06* (-3.40 to -2.86)		Decrease
Females	2000-2008	-2.27* (-2.67 to -1.43)	-3.02* (-3.18 to -2.86)	Decrease
Females	2008-2021	-3.47* (-3.87 to -3.24)		Decrease

**Figure 2:**
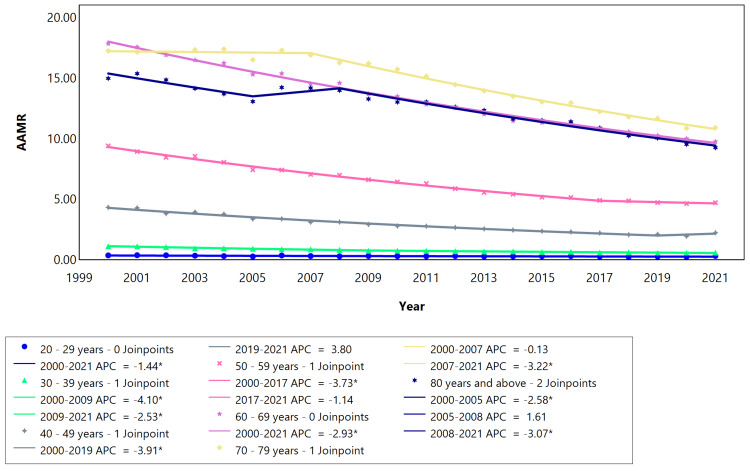
Cerebrovascular disease AAMR trend joinpoint analysis for the variable age for males during the period 2000-2021. *Significant at P < 0.05 level. AAMR = Age-adjusted mortality rate; APC = Annual percent change.

The AAMR between 2000 and 2021 for the category “males and females” ranged from 95 deaths/100,000 to 52 deaths/100,000 inhabitants (Figure 1). Males exhibited a higher mortality rate (108 deaths/100,000 inhabitants in 2000, declining to 63 deaths/100,000 in 2021) compared to females, who had a mortality rate of 83 deaths/100,000 in 2000, decreasing to 44 deaths/100,000 in 2021. Examining the AAMR of males across various age ranges revealed an overall decrease in mortality rates within all age groups. The most substantial reduction occurred in the 30-39-year age group from 2000 to 2009 (APC: -4.10, Table 2), while the smallest decline was observed in the 20-29-year age group spanning from 2000 to 2021 (APC: -1.44). The AAMR in the 70-79-year age group surpassed that of the 80 and above age group (see Figure 2). The 50 years and above (older age) age groups experienced a significant reduction in mortality rates, characterized by a steeper slope compared to the below 50 years (younger age) age groups (Figure 2). Additionally, there was a more stable and less steep change in mortality in the younger age over the years.

**Table 2:** Cerebrovascular disease AAMR trend joinpoint analysis for the variable age for males during the period 2000-2021. *Significant at P < 0.05 level. AAMR = Age-adjusted mortality rate; AAPC = Average annual percent change; APC = Annual percent change.

Age range (years)	Period (year)	APC (95% CI)	AAPC ( 95I% CI)	Interpretation
20-29	2000-2021	-1.44* (-2.07 to -0.86)	-1.44* (-2.07 to -0.86)	Decrease
30-39	2000-2009	-4.10* (-6.71 to -3.40)	-3.21* (-3.51 to -2.90)	Decrease
	2009-2021	-2.53* (-3.07 to -0.14)		Decrease
40-49	2000-2019	-3.91* (-4.72 to -3.62)	-3.20* (-3.81 to -2.92)	Decrease
	2019-2021	3.80 (-3.50 to 7.29)		Stable
50-59	2000-2017	-3.73* (-4.04 to -3.56)	-3.24* (-3.50 to -3.07)	Decrease
	2017-2021	-1.14 (-2.90 to -2.40)		Stable
60-69	2000-2021	-2.93* (-3.05 to -2.82)	-2.93* (-3.05 to -2.82)	Decrease
70-79	2000-2007	-0.13 (-0.76 to 0.74)	-2.20* (-2.37 to -2.02)	Stable
	2007-2021	-3.22* (-3.53 to -2.96)		Decrease
80 and above	2000-2005	-2.58* (-6.08 to -0.78)	-2.30* (-2.60 to -2.0)	Decrease
	2005-2008	1.61 (-5.20 to 3.22)		Stable
	2008-2021	-3.07* (-3.81 to -1.15)		Decrease

**Figure 3:**
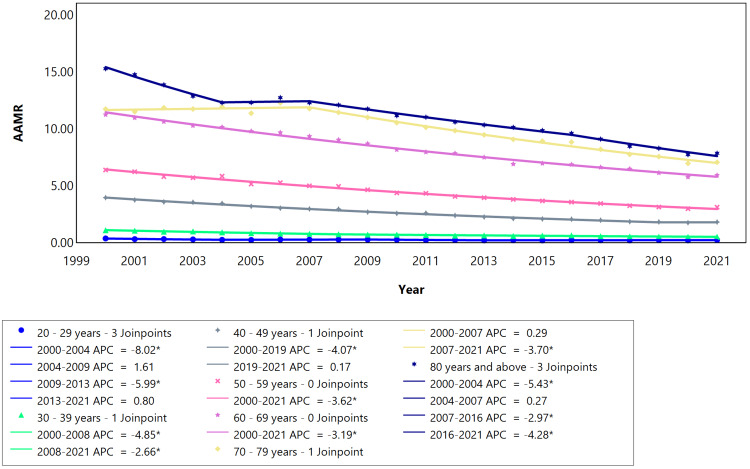
Cerebrovascular disease AAMR trend joinpoint analysis for the variable age for females during the period 2000-2021. *Significant at P < 0.05 level. AAMR = Age-adjusted mortality rate; APC = Annual percent change.

In the analysis of female age groups, those aged 80 and above experienced declining mortality from 2000 to 2004 and 2007 to 2016 (at a slower pace). A more pronounced decline occurred during 2016-2021 (Figure 3). The 70-79-year age group saw a significant decline starting in 2007. Females of age groups 50-59 and 60-69 had a constant regression throughout the analyzed years, as evidenced by the same APC and AAPC (Table 3). The mortality rate was lower in the younger age groups (below 50 years). Despite its lowest point being in the 20-29-year age group, there was a continuous decline observed throughout the study. This study highlighted the highest mortality rate within the elderly age group. The mortality rates were higher in the older age groups (50 years and above) compared to the younger population (below 50 years), as illustrated in Figure 3.

**Table 3:** Cerebrovascular disease AAMR trend joinpoint analysis for the variable age for females during the period 2000-2021. *Significant at P < 0.05 level. AAMR = Age-adjusted mortality rate; AAPC = Average annual percent change; APC = Annual percent change.

Age range (years)	Period (year)	APC (95% CI)	AAPC (95% CI)	Interpretation
20-29	2000-2004	-8.02* (-13.75 to -5.01)	-2.06* (-2.54 to -1.55)	Decrease
	2004-2009	1.61 (-0.49 to 7.14)		Stable
	2009-2013	-5.99*(-9.89 to -2.80)		Decrease
	2013-2021	0.80 (-0.67 to 5.48)		Stable
30-39	2000-2008	-4.85* (-7.20 to -3.96)	-3.50* (-3.82 to -3.18)	Decrease
	2008-2021	-2.66* (-3.17 to -1.45)		Decrease
40-49	2000-2019	-4.07* (-5.05 to -3.32)	-3.67* (-4.08 to -3.46)	Decrease
	2019-2021	0.17 (-4.05 to 2.76)		Stable
50-59	2000-2021	-3.62* (-3.80 to -3.45)	-3.62* (-3.80 to -3.45)	Decrease
60-69	2000-2021	-3.19* (-3.35 to -3.04)	-3.19* (-3.35 to -3.04)	Decrease
70-79	2000-2007	0.29 (-0.36 to 1.11)	-2.39* (-2.56 to -2.23)	Stable
	2007-2021	-3.70* (-4.00 to -3.44)		Decrease
80 and above	2000-2004	-5.43* (-7.25 to -4.51)	-3.30* (-3.52 to -3.15)	Decrease
	2004-2007	0.27 (-1.44 to 1.20)		Stable
	2007-2016	-2.97* (-3.38 to -2.40)		Decrease
	2016-2021	-4.28* (-6.62 to -3.53)		Decrease

**Figure 4:**
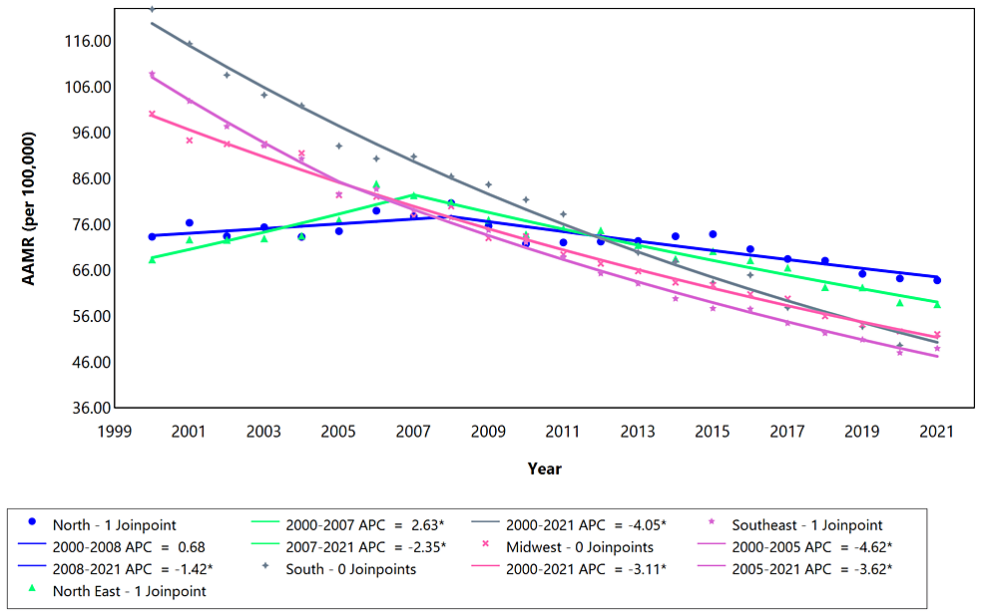
Cerebrovascular disease AAMR trend joinpoint analysis for the variable region of residence during the period 2000-2021. *Significant at P < 0.05 level. AAMR = Age-adjusted mortality rate; APC = Annual percent change.

All five macro-regions demonstrated statistically significant and downward AAPC values in mortality rates from 2000 to 2021, with the south region displaying the most prominent reduction and the north region the least (Table 4). The south, southeast, and midwest regions had a continuous decrease from 2000 to 2021 (Figure 4).

**Table 4:** Cerebrovascular disease AAMR trend joinpoint analysis for the variables state and region during the period 2000-2021. *Significant at P < 0.05 level. AAMR = Age-adjusted mortality rate; AAPC = Average annual percent change; APC = Annual percent change.

Region/State	Period	APC (95% CI)	AAPC (95% CI)	Interpretation
North	2000-2008	0.68 (-0.10 to 2.44)	-0.62* (-0.90 to -0.31)	Stable
	2008-2021	-1.42* (-2.10 to -1.02)		Decrease
Acre	2000-2006	6.28* (2.78 to 14.55)	0.23 (-0.57 to 1.25)	Increase
Acre	2006-2021	-2.09* (-3.46 to -1.18)		Decrease
Amapá	2000-2006	-9.03* (-21.25 to -3.82)	-1.86* (-2.93 to -0.63)	Decrease
Amapá	2006-2021	1.16 (-0.36 to 4.66)		Stable
Amazonas	2000-2021	-0.24 (-0.88 to 0.41)	-0.24 (-0.88 to 0.41)	Stable
Pará	2000-2004	-0.45 (-4.50 to 1.48)	-0.37* (-0.68 to -0.09)	Stable
Pará	2004-2008	5.34* (3.78 to 8.04)		Increase
Pará	2008-2011	-4.95* (-6.77 to -2.22)		Decrease
Pará	2011-2015	1.53 (-0.10 to 4.29)		Stable
Pará	2015-2021	-2.89* (-4.68 to -2.06)		Decrease
Rondônia	2000-2021	-2.58* (-3.05 to -2.16)	-2.58* (-3.05 to -2.16)	Decrease
Roraima	2000-2012	-4.19* (-7.10 to -2.49)	-0.65 (-1.60 to 0.32)	Decrease
Roraima	2012-2021	4.28* (1.36 to 10.15)		Increase
Tocantins	2000-2002	23.83* (13.34 to 30.45)	-0.79* (-1.24 to -0.34)	Increase
Tocantins	2002-2021	-3.08* (-3.41 to -2.79)		Decrease
Northeast	2000-2007	2.63* (1.61 to 4.14)	-0.72* (-0.98 to -0.42)	Increase
	2007-2021	-2.35* (-2.82 to -1.97)		Decrease
Alagoas	2000-2007	2.31* (0.56 to 6.20)	-0.68 (-1.21 to 0.00)	Increase
Alagoas	2007-2021	-2.14* (-3.24 to -1.50)		Decrease
Bahia	2000-2007	0.96 (-0.64 to 7.59)	-0.93* (-1.53 to -0.31)	Stable
Bahia	2007-2021	-1.86* (-4.07 to -1.28)		Decrease
Ceará	2000-2007	1.46* (0.12 to 4.22)	-1.01* (-1.39 to -0.62)	Increase
Ceará	2007-2021	-2.23* (-2.99 to -1.71)		Decrease
Maranhão	2000-2007	10.09* (7.43 to 14.11)	2.07* (1.47 to 2.88)	Increase
Maranhão	2007-2021	-1.71* (-2.68 to -0.86)		Decrease
Paraíba	2000-2007	7.33* (5.45 to 9.78)	0.00 (-0.44 to 0.52)	Increase
Paraíba	2007-2021	-3.48* (-4.20 to -2.84)		Decrease
Pernambuco	2000-2006	1.37* (0.70 to 2.41)	-2.03* (-2.28 to -1.83)	Increase
Pernambuco	2006-2009	-5.60* (-6.85 to -3.40)		Decrease
Pernambuco	2009-2016	-1.72 (-2.29 to 0.56)		Stable
Pernambuco	2016-2021	-4.26* (-6.62 to -3.23)		Decrease
Piauí	2000-2007	4.61* (3.18 to 6.59)	0.03 (-0.32 to 0.41)	Increase
Piauí	2007-2021	-2.18* (-2.75 to -1.69)		Decrease
Rio grande do Norte	2000-2007	3.42* (1.58 to 6.73)	-0.52 (-1.01 to 0.09)	Increase
Rio grande do Norte	2007-2021	-2.42* (-3.34 to -1.77)		Decrease
Sergipe	2000-2005	4.52* (1.22 to 12.72)	-1.08* (-1.68 to -0.19)	Increase
Sergipe	2005-2021	-2.77* (-3.72 to -2.15)		Decrease
Midwest	2000-2021	-3.11* (-3.31 to -2.93)	-3.11* (-3.31 to -2.93)	Decrease
Distrito Federal	2000-2014	-3.97* (-4.99 to -3.63)	-4.26* (-4.71 to -3.94)	Decrease
Distrito Federal	2014-2017	0.74 (-3.00 to 2.94)		Stable
Distrito Federal	2017-2021	-8.85* (-14.48 to -6.47)		Decrease
Goiás	2000-2021	-2.51* (-2.81 to -2.23)	-2.51* (-2.81 to -2.23)	Decrease
Mato grosso	2000-2021	-3.75* (-4.09 to -3.45)	-3.75* (-4.09 to -3.45)	Decrease
Mato grosso do sul	2000-2021	-3.19* (-3.60 to -2.80)	-3.19* (-3.60 to -2.80)	Decrease
Southeast	2000-2005	-4.62* (-6.65 to -3.77)	-3.86* (-4.05 to -3.65)	Decrease
	2005-2021	-3.62* (-4.38 to -2.25)		Decrease
Espírito Santo	2000-2003	-1.26 (-4.11 to 4.26)	-4.14* (-4.71 to -3.77)	Stable
Espírito Santo	2003-2021	-4.62* (-6.44 to -4.36)		Decrease
Minas gerais	2000-2021	-3.60* (-3.77 to -3.45)	-3.60* (-3.77 to -3.45)	Decrease
Rio de Janeiro	2000-2005	-5.68* (-7.83 to -4.82)	-4.00* (-4.23 to -3.80)	Decrease
Rio de Janeiro	2005-2008	-1.68* (-3.60 to -0.41)		Decrease
Rio de Janeiro	2008-2015	-5.21* (-7.44 to -4.58)		Decrease
Rio de Janeiro	2015-2021	-2.30 (-3.33 to 0.29)		Stable
São Paulo	2000-2005	-5.23* (-7.12 to -4.37)	-3.80* (-3.95 to -3.64)	Decrease
São Paulo	2005-2021	-3.35* (-3.55 to -3.05)		Decrease
South	2000-2021	-4.05* (-4.30 to -3.83)	-4.05* (-4.30 to -3.83)	Decrease
Paraná	2000-2021	-4.05* (-4.23 to -3.90)	-4.05* (-4.23 to -3.90)	Decrease
Rio grande do Sul	2000-2005	-4.85* (-7.62 to -3.53)	-4.13* (-4.42 to -3.88)	Decrease
Rio grande do Sul	2005-2010	-2.21 (-5.54 to 0.03)		Stable
Rio grande do Sul	2010-2021	-4.67* (-5.65 to -3.67)		Decrease
Santa catarina	2000-2002	-9.39* (-12.00 to -4.32)	-4.59* (-4.91 to -4.09)	Decrease
Santa catarina	2002-2021	-4.07* (-4.57 to -2.42)		Decrease

The south and midwest regions decreased at a stable rate (0 joinpoint) from 2000 to 2021 (Figure 4). The northeast regions initially exhibited an increase in the AAMR, followed by a decrease in the year 2007 (Figure 4). In the northeast, the joinpoint occurred in 2007 with the APC value changing from 2.63 to -2.35. In the southeast region, one joinpoint can also be seen in 2005, where the APC changed from -4.62 to -3.62. The highest mortality rate was initially recorded in the south region in 2000 (122 deaths/100,000 inhabitants), which decreased significantly in 2021 (51 deaths/100,000 inhabitants).

## Discussion

This study analyzed the AAMR trend for Brazil between 2000 and 2021. The variables considered were sex, region, and state. As per the World Health Organization, cerebrovascular diseases stand as the second leading cause of mortality and take precedence as the primary contributor to disability [11]. This underscores the urgency of maintaining up-to-date information regarding cerebrovascular disease trends, both as a gauge of strategy effectiveness and as a blueprint for future prevalence mitigation endeavors. Analyzing stroke data from the Global Burden of Disease (GBD) 2019 database exposed a significant upsurge in annual stroke occurrences and related fatalities spanning the period from 1990 to 2019. This escalation persisted despite notable declines in age-standardized rates (ASRs), particularly among individuals aged 70 and above, those with limited income, and those with elevated BMI [12].

Our investigation found a discernible decline in the trajectory of cerebrovascular disease mortality rates across the strata of males, females, and the combined group of males and females, throughout the span of 21 years (2000-2021). The decrease in mortality rates was more pronounced among females than males. Despite the higher mortality rates in the elderly (50 years and above) compared to the younger population (50 years and below), all age cohorts displayed a notable reduction in age-specific mortality rates. The entire spectrum of five macro-regions displayed a consistent reduction in mortality rates spanning the period from 2000 to 2021, with the south region registering the most substantial reduction and the north region manifesting the most modest decrease. It is worth noting that the most recent study encompassing the trends of cerebrovascular diseases within Brazil evaluated the years 2000-2018 and identified a declining mortality trend across all age groups [13], which aligns with our findings. This underscored the necessity for an up-to-date study, a gap that our research endeavored to fill.

Sex and age range

The mortality rates showed a downward trend in both males and females, with higher mortality rates recorded in males than females. This finding is in accordance with published literature, where it is established that higher mortality due to strokes occurs in men more than women [3]. Men are more likely to experience a stroke than women because they are more prone to alcoholism and smoking, as reported by the Stroke Association [14]. Understanding the declining mortality trend in both males and females is crucial in developing effective strategies for stroke prevention and intervention.

Our research found that the decline in mortality rate was more prominent after the years 2008-2021 (APC: -3.27) than 2000-2008 (APC: -2.17). This can be attributed to the improved awareness of the population on the risk factors of cerebrovascular diseases. A cross-sectional study on stroke awareness in Brazil showed that many respondents recognized smoking and hypertension as major risk factors for stroke [15]. There was a sharp decrease (41.8%) in smoking prevalence from 2006 to 2018 among the adult population of Brazil from 16.2% in 2006 to 9.3% in 2018 [16]. Hypertension is considered one of the main modifiable risk factors for stroke, and there has been a 6% reduction in the prevalence of hypertension among the Brazilian population in the past three decades [17]. This highlights the success of the efforts done in the country. Some of the efforts made toward the control of hypertension are the following: the state of São Paulo approved the public health policy called Hypertension and Diabetes Prevention and Control Public Health Policy in 1998, with the plan to reorganize the care provided to patients with hypertension and diabetes; the State of Goiás launched the CARMEN Project in 1999, with the objective to implement a set of health promotion actions to reduce the main noncommunicable disease risk factors, primarily the prevalence of hypertension; and the National Survey on non-communicable risk factors was implemented in the 27 states in 2003 [18].

Furthermore, Brazil has made efforts to reduce smoking rates. Results from the national health survey in 2019 showed that tobacco consumption reduced significantly in 2019 when compared to 2013. However, the goal of a reduction of 30% has not yet been achieved [19]. This progress can be credited to the Brazilian government for the implementation of various regulatory actions of the WHO Global Strategy on tobacco use in recent years. These actions include measures such as prohibiting smoking in public areas, regulating advertising and sponsorships, raising cigarette prices, and introducing cautionary labels and images on cigarette packages, among others [20].

The highest mortality rates occurred in older age groups (>50 years) for both males and females. In Brazil, increasing age is one of the main non-modifiable risk factors for ischemic stroke. Distinct mortality patterns emerged between the male and female cohorts; specifically, the female demographic exhibited the highest mortality rates in the 80 and above age group (Figure 3). Conversely, the male group displayed a more pronounced mortality rate in the age range of 70-79 years (Figure 2). Women tend to have higher incidence and mortality rates than men after the age of 85 [21]. This could be explained by the longer life expectancy of females compared to males and vascular changes as a result of old age [22].

Mortality from strokes among young adults is decreasing in Brazil [23]. This is in agreement with our findings, where the decline was observed in both sexes, in the age group 20-49 years. An additional noteworthy observation pertains to the age group 30-39 years for both males and females, where the previously sharp decline in mortality rates exhibited a reduced rate of decrease starting from 2008 in females and from 2009 in males. The prevalence of obesity has increased by 60% among those aged 25-34 years since 2006 in the Brazilian population, with the increase being more pronounced in females [24]. The way that obesity contributes to stroke is explained pathologically by various mechanisms, including diabetes mellitus, hypertension, accelerated atherosclerosis, atrial fibrillation, and obstructive sleep apnea [21]. This potentially leads to progressive atherosclerosis and/or thromboembolism that may result in arterial occlusion or rupture.

Region and state of residence

The states in the southeast region have greater economic importance and consist of more than 40% of the Brazilian population, including the most populous states of São Paulo and Rio de Janeiro, which had AAPCs of -3.80 and -4.00, respectively. Similar trends were recorded in cities such as Goias, Mato grosso, Mato grosso do sul, Minas gerais, and Parana. All these states demonstrated a steady decrease in mortality rate from 2000 to 2021. Some states located in the north and northeast regions (Ceará, Rio grande de Norte, Maranhāo, Paraíba, Sergipe, Alagoas, Pernambuco, and Piauí) recorded a significant increase in mortality rate throughout the years, followed by a decrease, as evidenced by the joinpoints between years 2005 and 2007 (Table 4). This is in accordance with the study by Vincens et al., which showed a similar increase in mortality due to stroke in these states between the years 2002-2009 [25]. However, the states that belong to the south, southeast, and midwest showed a downward trend in mortality rates during the studied years [25]. The decrease in mortality rates can be attributed to the implementation and expansion of the Brazilian unified public health system (SUS). Disparities in access to health in different states have been reported in the literature [25], which could also explain the trends observed in this study. The number of doctors per 1,000 inhabitants in the southeast is almost three times higher than that in the north [26], highlighting significant disparities and inequality among Brazilian states. The states exhibiting the highest levels of inequality are situated in the north and northeast regions, whereas those with the lowest levels are found in the south and southeast regions [27]. Income inequality can have adverse effects on population health, leading to heightened social stress and conflict, diminished trust, limited access to public goods, and reduced availability of healthcare services.

Limitations and strengths of the study

The utilization of secondary data, while often advantageous for its convenience and cost-effectiveness, comes with inherent limitations that should be acknowledged and considered when interpreting findings. Some of these limitations include the following: data quality and accuracy, especially with the classification of the causes of death (e.g., the ICD codes of deaths by cerebrovascular diseases could be entered incorrectly), and the possibility of incomplete information, leading to loss of information. Certain medical practitioners tend to classify deaths caused by cerebrovascular diseases as "other causes," leading to a potential underestimation of the actual figures and proportions of deaths attributed to cerebrovascular diseases. Another limitation inherent to this study is its exclusive emphasis on depicting trends in cerebrovascular mortality rates, without delving much into the underlying factors driving these trends. Subsequent research endeavors could delve into exploring the potential links between these mortality patterns and sociodemographic factors, including indicators such as income levels and healthcare spending. This is specifically important for Brazil, as it is one of the countries with substantial socioeconomic disparities.

In spite of the aforementioned limitations, our research is a novel and comprehensive study in this field. The main strength of the present study is the lengthy period it was able to cover (2001-2021). In addition, it covered the whole Brazil and analyzed information at various strata, including sex, age, state, and region of residence. There has not been a study of this magnitude done with the aim of investigating the trends of cerebrovascular diseases in Brazil. In addition, our study utilized a more thorough, complex, and convenient statistical approach to reach more reliable inferences. Our study has unveiled an updated information gap concerning the correlation between cerebrovascular disease mortality rates and socioeconomic indicators in Brazil. Consequently, we recommend that forthcoming research focuses on investigating this aspect.

## Conclusions

Our comprehensive analysis spanning two decades from 2000 to 2021 revealed a downward trend in cerebrovascular disease mortality rates across diverse demographic groups and macro-regions. Females experienced a more substantial reduction compared to males. Despite higher mortality rates among individuals aged 50 and above, all age groups displayed a marked decrease. The continuous decline in cerebrovascular disease mortality rates can be attributed to policy interventions aimed at enhancing healthcare delivery, increased awareness, and healthier diets and lifestyles. With regard to macro-regions, the south region demonstrated a more significant decrease as compared to the northern region. In Brazil, a more significant decline in cerebrovascular disease mortality rates could be achieved through increased focus on prevention measures and efforts toward mitigating disparities and inequalities between macro-regions.
